# An Assessment of the Allelopathic Impact of Sunflower on Seedlings of Spring Cereal Species Through Germination and Photosynthetic Performance

**DOI:** 10.3390/plants15050836

**Published:** 2026-03-09

**Authors:** Daiva Janusauskaite

**Affiliations:** Department of Plant Nutrition and Agroecology, Institute of Agriculture, Lithuanian Research Centre for Agriculture and Forestry, Instituto al. 1, Kėdainiai District, LT-58344 Akademija, Lithuania; daiva.janusauskaite@lammc.lt

**Keywords:** inhibitory effect, extract, *Helianthus annuus*, *Hordeum vulgare*, root length, shoot length, photosynthetic traits, *Triticum aestivum*

## Abstract

The effect of sunflower extract on the germination and development of weeds is investigated. However, knowledge about the effects of extracts on target plants is equally important. Investigations into the allelopathic relationship between sunflowers and cereals, which often make up 50–70% of crop rotations, still have many unanswered questions. This experiment aimed to investigate the allelopathic impact of sunflower (*Helianthus annuus* L.) as a donor plant for spring barley (*Hordeum vulgare* L.) and spring wheat (*Triticum aestivum* L.) through their germination and morphological parameters. The following three factors were studied: factor A—two growth stages of the donor plant; factor B—three parts of the donor plant; factor C—five concentrations (0%, 25%, 50%, 75% and 100%) of aqueous extracts of the plant donor. The extract concentration was the strongest factor influencing the germination of spring barley and spring wheat compared to the other two factors. The flowering sunflower extract inhibited the germination of the spring barley and spring wheat by 33–44% and 33–41%, respectively, more strongly than the ripe sunflower extract. According to the SE values, the allelopathic impact of extracts of sunflower parts on spring barley and spring wheat was as follows: L + S < R ˂ H and L + S < H ˂ R, respectively. The inhibitory effect of increasing concentration was determined on the SG, root/shoot length ratio, and SPAD values of both receptor plants.

## 1. Introduction

Allelopathy is a natural ecological process in which an organism produces biologically active compounds that affect the growth and development of other organisms [1]. Chemical exudates from some plants cause an adverse effect on the growth of neighboring plant species and thus affect normal growth in their natural environment [2,3]. The inhibitory properties of plants can be useful for weed management, but they can also be undesirable and damaging to other successive plants in crop rotation [3,4]. The way of action of exudates on plants involves many biochemical reactions. The allelocompounds of plants differ in their mechanism of action based on activities, such as germination and growth inhibition, respiratory disturbance, repellency, protein denaturation, and other effects, depending on the secreted compound and neighboring plants [5]. To apply a proper plant management strategy in crop rotation, it is very important to understand all the existing interactions between neighboring plants and between the pre-crop and the post-crop [6].

Sunflower is part of the group of the allelopathic plants. They produce a wide range of allelochemicals during the biosynthesis process, including phenolics, terpenoids, nitrogen-containing chemicals and many other chemical groups [4,7]. Recently, their cultivation areas have expanded to the Boreal region, where the adaptation of sunflowers to local soil and climatic conditions, yield potential and other properties in plant rotation are being studied [8,9,10,11,12,13].

The facility of plants to generate allelochemicals is often limited by the environment in which the plant is growing [1]. Environmental factors such as nutrient content in the soil, moisture regime, soil structure, and agricultural practices determine the concentration of allelochemicals in the plant [14]. Allelopathic plants can be applied in the strategy of plant protection for pest or weed management programs in various ways—as mulches or incorporated residues, such as catch crops, as cover crops, and as aqueous extracts when treating plants [15,16,17,18,19]. Because biotic and abiotic factors influence the expression of allelopathy [20], it is believed that sunflowers growing in new soil and climatic conditions may have a different allelopathic potential than those growing in warm climate zones. The allelopathic potential of sunflowers growing in their habitual growing zones has been explored in controlled conditions and field trials [21,22,23,24]. Sunflower residues adversely act on plant development and nutrient accumulation in some plants [9,21,23,24,25,26].

In warm climate zones, sunflower shows strong allelopathy for wheat (*Triticum aestivum*) [27,28,29,30,31], corn (*Zea mays*) [27], rice (*Oryza sativa*) [16], barley (*Hordeum vulgare* L.), lentil (*Lens culinaris* Medik.) [15], mung bean (*Vigna radiata* L. Wilczek) [22,32], lettuce (*Lactuca sativa*) [24], radish (*Raphanus sativus* L.) [33], bean, flax [26], and sponge gourd (*Luffa cylindrica*) [34].

The allelopathic potential of sunflowers grown in northern regions was studied for mustard (*Sinapis alba* L.) [10,11,35] and peas (*Pisum sativum* L.) [12,13]. However, not enough is known about the allelopathic potential of sunflowers grown in the boreal climate zone, and their impact on plants of the *Poaceae* family.

As the climate changes and the growing areas of sunflowers move to the boreal zone, many questions arise about the behavior of these plants in crop rotation and the impact on other plants. To diversify crop rotation, sunflowers with a deep root system are an excellent plant. However, before including them in a crop rotation, it is necessary to study how sunflowers affect plants under these climate and soil conditions. This study aimed to evaluate the allelopathic potential of sunflower (*Helianthus annuus* L.) grown in the boreal climate zone on the germination, development, and physiological parameters of spring wheat (*Triticum aestivum* L.) and spring barley (*Hordeum vulgare* L.).

## 2. Material and Methods

This factorial experiment was carried out using a randomized complete block design with three replications at the Institute of Agriculture, Lithuanian Research Centre for Agriculture and Forestry. There were three experimental factors: (1) factor A—donor plant growth stage (GS) in two levels: flowering sunflower (FS) and ripe sunflower (RS); (2) factor B—aqueous extract from different parts of sunflower plants in three levels: leaves and stems (L + S), heads (H), and roots (R); (3) factor C—concentration of extract in five levels: 0% (control), 25%, 50%, 75% and 100% *w*/*v*.

The sunflower (*Helianthus annuus* L., cultivar ‘Peredovick’) was a donor plant, and the spring barley (*Hordeum vulgare* L., cultivar ‘Freedway’) and spring wheat (*Triticum aestivum* L., cultivar ‘Colada’) were receptor plants. The parts of the sunflower for extracts were collected during the flowering stage and the full maturity stage from the experimental field of the Institute of Agriculture.

Donor plants were separated into different parts (roots, stems and leaves, and heads), which were crushed into small (10 mm) parts and air-dried. A cold-soaking method was used in the form of macerates by adding 100 g of dried plant material (L + S, H, and R) to 1000 mL of deionized water and soaking for 48 h at a temperature of 20–22 °C. The extract was filtered using Whatman No 1 filter paper. The filtrate was considered as 10% aqueous extract. By subsequent dilution with distilled water, aqueous extracts of 25%, 50%, 75% and 100% (based on volume) were prepared and stored in a refrigerator at 4 °C temperature until used. Deionized water was used as a control.

Grains of spring barley and spring wheat were washed with deionized water. Grains were germinated in a filter paper roll soaked with sunflower aqueous extracts as needed. Three replicates of 10 grains were performed for each treatment. Rolls of planted grains were placed in containers and kept in a 16/8 h light/dark cycle at 22 ± 2 °C temperature [36].

Seed germination (SG) was recorded at five, eight, eleven and fourteen days after sowing (DAS). A seed was considered germinated when the radicle was 1 mm long.

Root length and shoot length were measured using a ruler and the root/shoot length ratio was evaluated at 14 DAS. The root fresh mass (RFM) and shoot fresh mass (SFM) of spring barley and spring wheat seedlings were determined using an electronic balance with an accuracy of 0.001 g and the root/shoot fresh mass ratio was determined at 14 DAS.

Calculation of the root/shoot length ratio was based on the root and shoot length of the seedlings using the following formula:

Root/shoot length ratio = root length/shoot length(1)

Calculation of the root/shoot fresh mass ratio was based on the RFM and SFM of the seedlings using the following formula:

Root/shoot fresh mass ratio = RFM/SFM(2)

where RFM is the root fresh mass and SFM is the shoot fresh mass.

Donor plant (*Helianthus annuus* L., cultivar ‘Peredovick’) samples for the determination of bioactive compounds were collected from the sunflower grown at the Institute of Agriculture, Lithuanian Research Centre for Agriculture and Forestry. Leaves and stems (L + S), head (H), and root (R) samples were taken at the flowering stage (FS) and the ripe stage (RS). Before transportation to the laboratory, the samples were air-dried at room temperature (approximately 25 °C). The total flavonoid content and total polyphenolic compounds content (mg of rutin equivalent (RUE) g^−1^ of prepared sample) were determined according to spectrophotometric methods.

The second part of the study was conducted in the field. The soil of the experimental plot was Endocalcari–Epihypogleyic Cambisol. According to agrochemical indicators, the soil was close to a neutral reaction (pHKCl 6.3–6.8) (potentiometrically), medium rich in humus (2.2–2.4%) (Tyurin method), medium rich in available phosphorus (128–152 mg kg^−1^) (A-L method) and rich in available potassium (162–195 mg kg^−1^) (A-L method). On 14 DAS rolls, the seedlings of the studied plants were transplanted into the field according to a scheme analogous to the laboratory experiment. The study was performed in three replications, 10 plants in each. The distance between rows was 12 cm, and between plants within a row was 5 cm. The physiological parameters and gas exchange parameters were assessed at the same time, on randomly selected leaves of 10 plants per line, from 10 am until 2 pm (local time) on clear days three times per growing season. The measurements were carried out in barley at the stem elongation (BBCH 30–31), at the flag leaf stage (BBCH 39) and the end of the heading (BBCH 59), and in spring wheat at the beginning of stem elongation (BBCH 30), at the booting stage (BBCH 41–43) and at full flowering stage (BBCH 65). Relative chlorophyll content (SPAD index) was estimated with a SPAD 502 (Minolta Camera Co., Ltd., Osaka, Japan). The maximum quantum efficiency of PSII photochemistry (Fv/Fm) was measured using a multi-function pulse-modulated handheld chlorophyll fluorometer (model OS 30p; Manufacturer: Opti-Sciences, Inc., Hudson, NH, USA). Fv/Fm was directly read after 20 min dark adaptation on the chlorophyll fluorometer [37]. The actinic light intensity was 1000 µmol m m^−2^ s^−1^, with a modulation intensity of two arbitrary units. Leaf gas exchange measurements were performed using a portable infrared gas analyzer (SRS-1000) (ADC BioScientific Ltd., Hoddesdon, UK). The measurements were made on three randomly selected plants per plot on the first fully expanded leaf from the top. Photosynthetic parameters such as photosynthetic rate (A) (µmol m^−2^ s^−1^), transpiration rate (E) (mmol m^−2^ s^−1^), stomatal conductance (gs) (mol m^−2^ s^−1^) and intercellular CO_2_ concentration (Ci) (µmol mol^−1^) were recorded.

The inhibitory rate (IR) was used to indicate the allelopathic effects of aqueous leaf extracts on morphological parameters and physiological characteristics:

IR = 1 − C/T, if T ≥ C(3)

IR = T/C − 1, if T ˂ C(4)

where T is the treatment value and C is the control value [38].

An IR < 0 reflects a negative allelopathic effect, i.e., denotes the inhibition of sunflower extracts on tested parameters, and an IR > 0 indicates a promoting effect on the parameters of receptor plants. The synthetical allelopathic effect index (SE) was calculated as the arithmetic mean value of the IR [38].

The data of SG, root/shoot length ratio, and root/shoot fresh mass ratio of both spring barley and spring wheat were analyzed according to a three-way analysis of variance (ANOVA) model: two growth stages of sunflower (factor A), three plant parts of donor plant (factor B), and five concentrations of sunflower aqueous extracts (factor C). The data of physiological traits and gas exchange parameters of spring wheat were analyzed according to a three-factor ANOVA model; these indicators for spring barley were analyzed by one-way ANOVA, because factors of growth stage and plant part of the donor plant were found to be non-significant.

Significant differences among treatments were determined according to Fisher’s least significant difference (LSD) test at the probability levels of *p* ≤ 0.05 and *p* ≤ 0.01. The statistical analysis was done using software ANOVA from the statistical software package Selekcija, version 4.0 [39].

## 3. Results

### 3.1. Dynamics of Seed Germination and Seedling Growth of Spring Barley and Spring Wheat Under the Influence of Sunflower Extracts

The results of the ANOVA showed that sunflower aqueous extracts had different effects on the germination of spring barley and spring wheat during four evaluations—5 DAS, 8 DAS, 11 DAS and 14 DAS (Table 1).

In spring barley, in all four assessments, three-way ANOVA revealed that seed germination (SG) was significantly (*p* ≤ 0.01) influenced by sunflower growth stages (GSs) (factor A), sunflower aqueous extract concentration (factor C), and their interaction (A × C). Plant part (factor B) was found to have a significant (*p* ≤ 0.01) effect on the SG of spring barley only at 11 DAS and 14 DAS. According to the mean squares expressed as a percentage of the sum of squares, the extract concentration (factor C) was the main factor responsible for 62–68% of the total variability in the SG of spring barley. Sunflower GS (factor A) and A × C interaction governed a similar percentage of variation—4–8% and 4–5%, respectively.

In spring wheat, in all four assessments, ANOVA showed that SG was significantly (*p* ≤ 0.05, *p* ≤ 0.01) influenced by all factors and their interactions (Table 1). According to the mean squares expressed as a percentage of the sum of squares, the aqueous extract concentration (factor C) was the main factor that determined 33–37% of the total variability of SG of spring wheat. Sunflower GS (factor A) explained 14–20%, and plant part (factor B) 11–18% of the total variability of SG.

**Figure 1:**
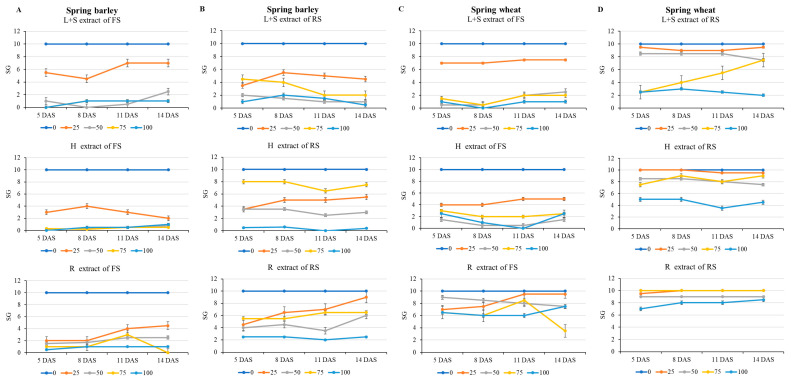
Allelopathic impact of different concentrations of the extracts of sunflower parts on seed germination (SG) of spring barley. (**A**)—extract of flowering sunflower (FS), (**B**)—extract of ripe sunflower (RS), and spring wheat. (**C**)—extract of flowering sunflower (FS), (**D**)—extract of ripe sunflower (RS) 5, 8, 11 and 14 days after sowing (DAS). L + S—leaves and stems; H—heads; R—roots. The error bars show SE.

The dynamics of the SG measured during the four assessments (5 DAS, 8 DAS, 11 DAS and 14 DAS) are provided in Figure 1. The FS extract (Figure 1A,C) had an inhibitory effect on the germination of both receptor plants compared to the RS extract (Figure 1B,D). Comparing FS vs. RS, the SG of spring barley and spring wheat was suppressed by 33–44% and 33–41%, respectively, throughout all assessments. The effect of plant part extract on receptor plants’ SG was different. On average, the inhibiting effect of aqueous extracts of sunflower parts on both spring barley and spring wheat was as follows: L + S ˂ H ˂ R.

The results showed that compared to the first assessment, at the final evaluation, the R extract resulted in 18% higher SG of spring barley. Meanwhile, in spring wheat, SG increase (+13%) was found only with L + S extract. The SG of both spring barley and spring wheat decreased with increasing extract concentration in most cases: 0% < 25% < 50% < 75% < 100%.

**Figure 2:**
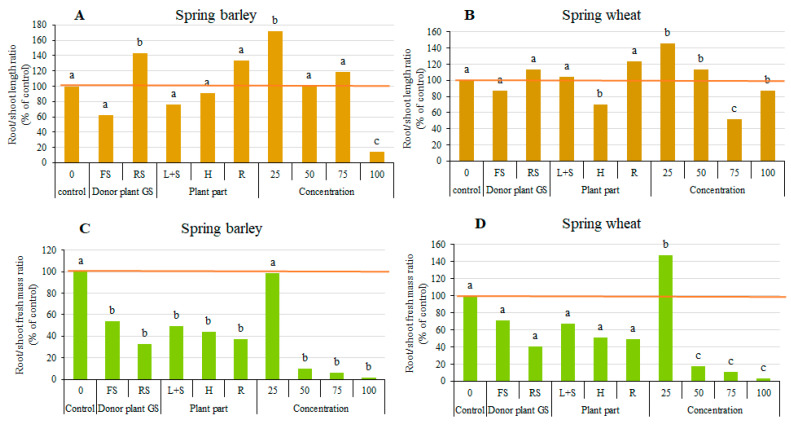
Impact of donor plant growth stage, plant part and extract concentration of sunflower plants on root/shoot length ratio of spring barley (**A**) and spring wheat (**B**), and on root/shoot fresh mass ratio of spring barley (**C**) and spring wheat (**D**), 14 DAS. FS—flowering sunflower; RS—ripe sunflower; L + S—leaves and stems; H—heads; R—roots. Different letters denote a statistically significant difference (at *p* ≤ 0.05 according to LSD) among treatments.

Figure 2 shows the present root/shoot length ratio in percent of the control (Figure 2A,B), and root/shoot fresh mass ratio in percent of the control (Figure 2C,D). The data averaged across plant part and extract concentration revealed that RS extract significantly increased the root/shoot length ratio of spring barley by 42.9%, in comparison with the control treatment (Figure 2A). Differences in the root/shoot length ratio between plant part treatments were insignificant. The root/shoot length ratio of spring barley increased by 71% and decreased by 86% by applying 25% and 100% extracts, respectively, and differences were significant (*p* ≤ 0.01), in comparison with the 0% concentration.

In spring wheat, differences in the root/shoot length ratio applying FS and RS were insignificant (Figure 2B). H extract significantly (*p* ≤ 0.05) decreased the root/shoot length ratio of spring wheat by 30%, whereas R extract tended to increase this parameter. Low (25%) extract concentration significantly (*p* ≤ 0.05) increased the root/shoot length ratio of spring wheat; however, the index decreased with increasing concentration, and when applying 75%, the difference was significant, compared to the control.

The response of the root/shoot fresh mass ratio in both receptor plants to aqueous extracts of FS and RS and parts of the sunflower was similar, and, in comparison to the control treatment, significantly (*p* ≤ 0.05) decreased in spring barley (Figure 2C), but differences were not significant in spring wheat (Figure 2D). The 25% extract significantly (*p* ≤ 0.05) increased the root/shoot fresh mass ratio in spring wheat (by 47%). The index significantly (*p* ≤ 0.05) decreased with increasing concentration in both spring barley and spring wheat.

**Figure 3:**
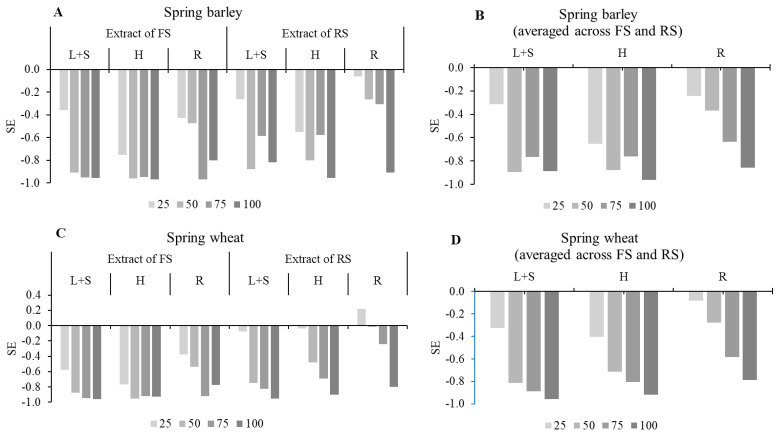
Allelopathic synthetic effect (SE) of sunflower aqueous extracts on seed germination 14 DAS, and all tested morphological parameters (root length, shoot length, root fresh mass, and shoot fresh mass). ((**A**)—effect of FS and RS extracts on spring barley; (**B**)—effect averaged across FS and RS on spring barley; (**C**)—effect of FS and RS extracts on spring wheat; (**D**)—effect averaged across FS and RS on spring wheat). FS—flowering sunflower; RS—ripe sunflower; L + S—leaves and stems; H—heads; R—roots.

The synthetical allelopathic effect index (SE) evidenced the allelopathic effect of the tested factors on SG 14 DAS and all tested morphological parameters, including the root length, shoot length, root fresh mass, and shoot fresh mass (Figure 3A–D). The SE showed that sunflower aqueous extracts had an inhibitory effect on the mentioned parameters in all cases, with one exception, when R extract at 25% concentration of ripe sunflower (RS) revealed the stimulatory effect and the SE was positive (Figure 3C). The SE increased with increasing extract concentration. Averaged across FS and RS, R extract revealed the lowest inhibitory effect on both receptor plants. According to SE values, the trend R ˂ L + S < H was for spring barley and the trend R ˂ H <L + S was for spring wheat.

It was found that the content of biologically active compounds in different parts of flowering and ripe sunflowers varied (Table 2). In flowering sunflowers (FSs), the highest content of total polyphenolic compounds and total flavonoids were found in H, 32.5 RUE g^−1^ and 39.3 mg RUE g^−1^, respectively. Meanwhile, in L + S and R, at the flowering growth stage, polyphenolic compounds were 46.5% and 67% lower, respectively, and total flavonoids were 58.5% and 76.1% lower, respectively, compared to H. When the donor plants reached ripening (RS), the content of biologically active compounds in the plant parts was distributed differently. It was found that most of them were in R; the content of total polyphenolic compounds was 17.2 mg RUE g^−1^ and total flavonoids was 19.0 mg RUE g^−1^, or 64% and 100.2% higher, compared to R in the FS.

Compared to data in the FS, in the L + S and H of ripened sunflowers (RSs), the total content of polyphenolic compounds was 27.0% and 66.2% lower, respectively, and the total content of flavonoids was 44.8% and 80.0% lower.

### 3.2. Impact of the Extracts on Physiological Parameters and Gas Exchange Indices of Target Cereals

Grains germinated in rolls were transplanted to the field after the last evaluation of morphological indicators. Due to the inhibitory effect of the extracts during germination, not all spring barley seedlings were successfully established in the field. Therefore, we analyzed the data of the physiological indicators of barley by one-factor ANOVA (Table 3). R extract at 25% concentration from the FS demonstrated the highest inhibitory effect on the chlorophyll index (SPAD) and significantly (*p* ≤ 0.05) inhibited SPAD by 11%; however, the effect of 25% R extract on the maximum quantum efficiency of PSII photochemistry (Fv/Fm) and intercellular CO_2_ concentration (Ci) was opposite: it significantly (*p* ≤ 0.05) stimulated the parameters by 3% and 21%, respectively. R 50% extract significantly (*p* ≤ 0.05) stimulated the SPAD, transpiration rate (E), and stomatal conductance (gs), respectively, by 13%, 55% and 54%, compared to the control treatment (0%).

Under RS application, R 25% extract significantly stimulated the SPAD (by 15%); L + S 25% extract and H 75% extract significantly enhanced the Fv/Fm of spring barley by 4%.

Three-way ANOVA showed that plant part (factor B), extract concentration (factor C), their interaction (B × C), and the interaction of the donor plant’s GS and extract concentration (A × C) had a significant (*p* ≤ 0.05, *p* < 0.01) effect on the SPAD of spring wheat (Table 4). The extract concentration was the main factor that determined 30.3% of the SPAD differences between treatments.

The Fv/Fm and E were significantly (*p* ≤ 0.05) influenced only by extract concentration, explaining 4.6% and 14.9%, respectively, of the total variability of values.

All tested factors and their interactions had a significant (*p* ≤ 0.05, *p* < 0.01) effect on the A, gs and Ci of spring wheat in most cases. The interaction of plant part and extract concentration (B × C) was responsible for the largest part of A, gs and Ci data, respectively, 14.0%, 20.6% and 21.4%.

The inhibitory rate (IR) exposed the allelopathic effect of the tested factors on the physiological traits of the target plants (Table 5). It was found that, in spring barley, sunflower extracts at different concentrations had stimulating effects on the SPAD, Fv/Fm, E, gs and Ci in 60%, 70%, 70%, 70% and 70% of the cases, respectively; however, the A was inhibited in 80% of the cases.

In spring wheat, the tested extracts revealed a stimulating effect on the Fv/Fm, A, E, gs and Ci in 96%, 63%, 88%, 67% and 50% of the cases, respectively. Meanwhile, the SPAD was inhibited in 92% of all the tested cases.

**Figure 4:**
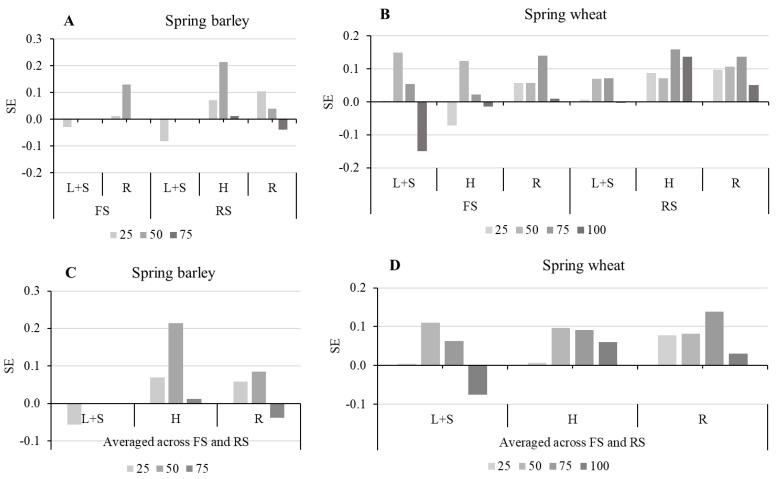
Allelopathic effects (SE) of donor plant growth stage, plant part, sunflower aqueous extract concentration on physiological traits of spring barley (**A**,**C**) and spring wheat (**B**,**D**). SE was calculated as the average inhibitory rate (IR) of all indicators with each treatment. FS—flowering sunflower; RS—ripe sunflower; L + S—leaves and stems; H—heads; R—roots.

Six indicators (SPAD, Fv/FM, A, E, gs and Ci) were used to estimate the allelopathy SE value to define the total allelopathic potency of the sunflower extract at different concentrations on the target spring barley and spring wheat (Figure 4). The SE in spring barley, applying FS and RS extracts, was found to be positive and stimulating (0.04 and 0.05, respectively) (Figure 4A). On average, across FS and RS extracts, the trend of the SE was as follows: L + S < R ˂ H (−0.06, 0.03 and 0.10, respectively) (Figure 4C).

Regarding the SE in spring wheat, when applying both FS and RS extracts, the extract from different plant parts had minor, but positive and stimulating allelopathic, effects (under FS + 0.03, under RS + 0.08, respectively) (Figure 4B). On average across FS and RS extracts, the trend of the SE in spring wheat was as follows, L + S < H ˂ R, and SE ranged from 0.03 to 0.08. The highest stimulating effect on physiological traits was R extract (SE was 0.08) (Figure 4D).

## 4. Discussion

Seed germination (SG) is the first relevant phase for the growth and development of a plant and a fundamental factor in reaching a desirable crop density. Plants with allelopathic potential have a phytotoxic influence on SG [40,41] and subsequent growth and development of other plants [30,42]. The allelopathic effect can be in two directions—inhibiting [43,44,45] or stimulating the germination and growth of recipient plants [46,47]. The study of Muhammad and Majeed [27] showed that sunflower extract significantly reduced the SG and seedling development of wheat and maize. In another study on the allelopathic activity of sunflower [8], it was found that sunflower aqueous extract inhibited the germination of mustard seeds and the allelopathic potential of the two investigated sunflower varieties was different. The inhibitory effect of sunflower extracts on the SG of wheat, wild barley, mustard, and radish was evaluated, and there was found that the inhibition increased with increasing concentration of the extract [29,33,35,43]. Our results revealed that the SG of both receptor plants—spring barley and spring wheat—decreased with the increasing concentration of sunflower aqueous extract. We found that barley was more sensitive to sunflower extracts in terms of SG than spring wheat. Our experiments are in line with studies of the allelopathic activities of catnip (*Nepeta meyeri* Benth.) root and shoot extracts on barley and wheat [48]. They found that barley reacted more sensitively to extracts of *Nepeta meyeri* than wheat, and leaf and root extracts of *Nepeta meyeri* provoked a more intense suppressive impact on the SG and seedling development of barley than of wheat.

Different organs of allelopathic crops accumulate different amounts of allelopathic compounds [2,46,49]. Extracts from different parts of the plant may differ in allelopathic effectiveness [11,30]. The results of the current study show that, on average, the inhibiting effect of aqueous extracts of sunflower parts on receptor plants was as follows: L + S ˂ H ˂ R. Rigon et al. [33] found that the allelopathic impacts of sunflower extracts rise in the following order: root < stem < leaf. Similar data were obtained in an experiment with wheat and maize as receptors: it was found that the leaf extract had the strongest inhibitory impact on SG [27].

We found that the FS extract revealed an inhibitory effect on the SG of receptor plants, in comparison to the RS extract. The inhibitory effect of FS extract was similar for both recipient plants. Our data are consistent with previous studies where differences in allelopathic potential between the growth stages of plant donors were revealed as sunflower [24], rape, turnip and mustard [50]. The amount of allelochemical substances in the above-ground part of plants decreases with aging, and in the roots, it increases [24,51].

Root/shoot rate is one of the keys to plants’ capability to compensate for limiting reserves in their surroundings [52]. This index can be exchanged in response to numerous circumstances, such as nutrient availability in the soil or the neighborhood of other plants that have allelopathic properties [49,52,53]. Sunflower leaf, stem, flower and root extracts inhibited seed germination, root and hypocotyl elongation and mass accumulation of wild barley (*Hordeum spontaneum*) [43]. We found that the root/shoot length ratio of spring barley significantly increased in RS extract treatment. However, the difference in the indicator of spring wheat between FS and RS variants was insignificant.

Biologically active allelopathic compound phenols produce suppressive effects on plant growth and frequently cause root morphological alteration [20]. In the current study, the low concentration (25%) had a positive influence on the root/shoot length ratio of both target crops, and, on the contrary, higher concentrations revealed another type of influence — a negative influence on the values of the indicator, compared to the control. In the investigation of the allelopathic possibility of ajowan (*Carum copticum*) extracts in different concentrations on the morphological parameters of wheat (*Triticum sativum*), barley (*Hordeum vulgare*), corn (Zea maize), and safflower (*Carthamus tinctorius* L.) seedlings, it was found that the shoot/root length ratio gradually decreased with increasing concentration of extract [54]. In contrast to our data, Far et al. [54] did not determine the stimulatory effect of the lowest concentrations on the root/shoot length ratio.

In the current study, low (25%) extract concentration significantly stimulated the root/shoot fresh mass ratio in both recipient crops. However, concentrations higher than 25% (50%, 75%, and 100%) significantly (*p* ≤ 0.05) inhibited the mentioned index. As other studies confirm [19,30,38], the factors that exert influence on the efficiency of allelopathic compounds are recipient plant, extract concentration, and duration of inhibition. The extracts of the donor plant disturb nutrients and water absorption by seedling roots due to the presence of phenolic compounds, which may ultimately diminish photosynthesis and seedlings weights [55].

The allelopathic effects and concentration of allelochemicals differ among the organs of the allelopathic plant [3,43]. In our study, significant differences between variants of plant parts were determined only once—H extract significantly reduced the root/shoot length ratio of spring wheat. Oueslati et al. [56], investigating the effect of different plant parts, found that leaf extracts had the most allelopathic effect in reducing the root and shoot length of the recipient plant, compared with root and stem extracts. Similar patterns were found in a study with wheat and allelopathic weeds on each other’s intercropping: the fresh and dry weight of wheat shoots showed a significant decrease at different ratios of wheat and allelopathic weeds, compared to the control [57].

Chlorophyll molecules are a fundamental part of pigment–protein complexes placed in the photosynthetic membranes and carry out a role of major importance in photosynthesis. The chlorophyll content and Fv/Fm seem to be useful criteria to assess allelopathic stress in plants [58]. The synthesis of chlorophyll in mung bean *(Vigna mungo)* was inhibited as the concentrations of black pepper (*Piper nigrum*) extract increased [59]. It should be noted that the plant donor samples collected at different growth stages for extract production differed in allelopathic nature. We found that a significant decrease in the SPAD of spring barley was noted when only applying 25% R extract from the FS. Also, a significant stimulating effect of the SPAD was established using 50% R extract from the FS and 25% R extract from the RS. In spring wheat, H extract showed the strongest inhibitory effect on SPAD values. The data, averaged across donor plant growth stages and plant parts, revealed that, as the extract concentrations increased, the SPAD in wheat leaves consistently decreased. These data are consistent with trends revealed in a weed–wheat intercrop, where chlorophyll decreased significantly with increasing weed density [57]. In a study that investigated the effect of allelopathic seed meals on the weed’s physiological properties, it was also found that, compared to the control, the SPAD significantly decreased with increasing amounts of allelopathic plant seed meal incorporated into the soil [60].

## 5. Conclusions

The extracts of sunflowers deleteriously affected the germination of spring barley and spring wheat. FS extract inhibited germination of both receptor plants, with a stronger effect than RS extract during the first evaluations—5 DAS and 8 DAS. Also, FS extract showed a stronger inhibitory effect on physiological properties than RS extract.

The inhibitory effect of increasing concentration was determined on the SG, root/shoot length ratio, root/shoot fresh mass ratio and SPAD values of both receptor plants. The inhibitory effect of increasing concentrations on SPAD values was greater in spring wheat than in barley.

Regarding SE values on physiological parameters of spring barley and spring wheat, averaged across FS and RS extracts, the trend was as follows: L + S < R ˂ H and L + S < H ˂ R, respectively.

The insertion of plants with allelopathic properties, especially those new to those climatic zones, in crop rotation can have a noticeable effect not only on weed control, but also on cultivated plants. The results of this study improve our understanding of the behavior of sunflowers in crop rotation and their influence on other plants.

## Figures and Tables

**Table 1:** Analysis of variance of donor plant growth stages (GSs), plant part, aqueous extract concentration, and their interaction on seed germination of spring barley and spring wheat (the table shows the sources of variation and probability (*p*) for the *F*-test of each factor and its interactions).

Action and Interaction	DF	5 DAS	8 DAS	11 DAS	14 DAS
Spring barley					
Donor plant GS (A)	1	21.13 **	37.94 **	19.80 **	31.12 **
Plant part (B)	2	1.28	0.70	3.44 **	7.00 **
Extract concentration (C)	4	76.46 **	69.96 **	86.14 **	88.98 **
A × B	2	1.39	0.96	2.78	10.56 **
A × C	4	6.14 **	5.78 **	4.93 **	6.91 **
B × C	8	1.12	1.03	1.73	2.38 **
A × B × C	8	1.48	1.28	1.16	1.74
Spring wheat					
Donor plant GS (A)	1	114.91 **	169.34 **	101.55 **	85.57 **
Plant part (B)	2	58.17 **	57.68 **	64.68 **	29.58 **
Extract concentration (C)	4	71.74 **	69.43 **	68.26 **	42.77 **
A × B	2	10.07 **	12.77 **	14.25 **	3.90 *
A × C	4	12.45 **	13.59 **	9.53 **	12.07 **
B × C	8	10.55 **	8.67 **	6.76 **	5.45 **
A × B × C	8	5.03 **	3.80 **	2.34 **	1.25

** and * indicate statistical significance at *p* ≤ 0.01 and *p* ≤ 0.05, respectively.

**Table 2:** Content of biological active compounds in sunflower parts.

	Total Polyphenol Content, mg RUE g^−1^		Total Flavonoid Content, mg RUE g^−1^	
	FS	RS	FS	RS
L + S	17.4	12.7	16.3	9.0
H	32.5	11.0	39.3	7.9
R	10.5	17.2	9.4	19.0

FS—flowering sunflower, RS—ripe sunflower, L + S—leaves and stems; H—heads; R—roots.

**Table 3:** Effects of donor plant growth stage, plant part, and sunflower aqueous extract concentration on physiological traits of spring barley in the field (averaged three measurements).

Donor Plant GS	Plant Part	Concentration, % *w*/*v*	SPAD	Fv/Fm	A	E	gs	Ci
		0	37.6 a	0.692 a	5.28 a	0.56 a	0.037 a	239 a
FS	L + S	25	36.7 a	0.687 a	5.26 a	0.51 a	0.033 a	240 a
	R	25	33.5 b	0.711 b	5.31 a	0.49 a	0.042 a	288 b
		50	42.4 c	0.688 a	4.73 a	0.87 b	0.057 b	261 a
		Average of FS	37.6	0.695	5.15	0.61	0.042	257
		*p*	**	**		*		*
		0	37.6 a	0.692 a	5.28 a	0.56 a	0.037 a	239 a
RS	L + S	25	34.5 a	0.718 b	4.45 a	0.43 a	0.030 a	275 a
	H	25	37.7 a	0.701 a	5.12 a	0.79 a	0.043 a	235 a
		50	41.3 a	0.694 a	4.82 a	0.80 a	0.063 b	282 a
		75	34.8 a	0.720 b	3.84 a	0.79 a	0.040 a	242 a
	R	25	43.4 b	0.701 a	4.06 a	0.86 a	0.047 a	279 a
		50	40.1 a	0.683 a	2.68 a	0.78 a	0.040 a	360 b
		75	41.3 a	0.706 a	2.99 a	0.69 a	0.033 a	238 a
		Average of RS	39.5	0.703	4.42	0.73	0.040	273
		*p*	**	**				*

GS—growth stage; SPAD—chlorophyl index; Fv/Fm—maximum quantum efficiency of PSII photochemistry; A—photosynthetic rate; E—transpiration rate; gs—stomatal conductance; Ci—intercellular CO_2_ concentration; FS—flowering sunflower; RS—ripe sunflower; L + S—leaves and stems; H—heads; R—roots. Different letters in columns and *, ** indicate significant (at *p* ≤ 0.05 and *p* ≤ 0.01, respectively) differences between treatments according to Fisher’s least significant difference (LSD) test.

**Table 4:** Effects of donor plant growth stage, plant part, sunflower aqueous extract concentration on physiological traits of spring wheat in the field (average of three measurements).

Donor Plant	Plant	Concentration,	SPAD	Fv/Fm	A	E	gs	Ci
GS	Part	% *w*/*v*						
FS			38.8 a	0.668 a	1.68 a	0.98 a	0.11 a	337 a
RS			38.6 a	0.671 a	2.21 b	1.07 a	0.05 b	321 b
	L + S		39.1 a	0.673 a	1.75 a	1.01 a	0.13 a	330 a
	H		38.1 b	0.670 a	1.91 a	1.05 a	0.05 b	331 a
	R		39.1 a	0.666 a	2.16 b	1.03 a	0.05 b	326 a
		0	41.7 a	0.658 a	1.54 a	0.85 a	0.05 a	333 a
		25	41.5 a	0.665 a	2.04 b	0.95 a	0.05 a	313 b
		50	38.4 b	0.673 b	2.21 b	1.11 b	0.06 a	333 a
		75	37.2 c	0.673 b	2.25 b	1.19 b	0.15 b	332 a
		100	34.9 c	0.679 b	1.67 a	1.03 b	0.10 b	330 a
Contribution (% of the sum of squares) of donor plant GS, plant part, aqueous extract concentration and their interaction and significance								
Growth stage (Factor A)			0.1	0.2	8.0 **	2.4	6.1 **	4.8 **
Plant part (Factor B)			0.8 *	0.5	3.4 *	0.3	11.5 **	0.3
Concentration (Factor C)			30.3 **	4.6 **	9.5 **	14.9 **	12.3 **	5.2 *
A × B			0.8	1.0	2.1	1.1	13.5 **	0.1
A × C			1.7 *	0.7	9.0 **	3.1	8.9 **	5.5 *
B × C			3.8 **	0.8	14.0 **	5.5	20.6 **	21.4 **
A × B × C			2.1	1.0	10.7 **	7.6	19.3 **	15.9 **

FS—flowering sunflower; RS—ripe sunflower; L + S—leaves and stems; H—heads; R—roots; Different letters in columns and *—significant at *p* ≤ 0.05; **—significant at *p* ≤ 0.01.

**Table 5:** The inhibitory rate (IR) for physiological traits of target plants.

Donor Plant GS	Plant Part	Concentration	IR_SPAD_	IR_Fv/Fm_	IR_A_	IR_E_	IR_gs_	IR_Ci_
Spring barley								
FS	L + S	25	−0.02	−0.01	0.00	−0.09	−0.09	0.00
	R	25	−0.12	0.03	0.00	−0.13	0.12	0.17
		50	0.12	−0.01	−0.10	0.36	0.35	0.09
RS	L + S	25	−0.09	0.03	−0.16	−0.23	−0.18	0.13
	H	25	0.01	0.01	−0.03	0.29	0.15	−0.02
		50	0.07	0.00	0.33	0.30	0.42	0.15
		75	−0.08	0.04	−0.27	0.30	0.08	0.01
	H	25	0.13	0.01	−0.23	0.35	0.21	0.14
		50	0.06	−0.01	−0.49	0.28	0.08	0.34
		75	0.09	0.02	−0.43	0.19	−0.09	0.00
Spring wheat								
FS	L + S	25	−0.01	−0.01	−0.17	0.15	0.03	0.02
		50	−0.06	0.02	0.36	0.35	0.26	−0.03
		75	−0.09	0.03	−0.02	0.14	0.18	0.09
		100	−0.24	0.04	−0.44	−0.02	−0.21	−0.01
	H	25	−0.01	0.01	−0.03	−0.24	−0.07	−0.08
		50	−0.16	0.04	0.23	0.27	0.36	0.00
		75	−0.10	0.03	−0.29	0.25	0.13	0.12
		100	−0.18	0.04	−0.18	0.15	0.07	0.03
	R	25	0.07	0.01	−0.07	0.21	0.07	0.06
		50	−0.06	0.01	0.39	0.05	−0.07	0.02
		75	−0.09	0.01	0.43	0.28	0.26	−0.05
		100	−0.17	0.02	0.26	0.03	−0.07	0.00
RS	L + S	25	−0.01	0.03	0.55	−0.04	−0.21	−0.28
		50	0.03	0.04	0.33	0.14	−0.07	−0.03
		75	−0.16	0.03	−0.26	0.37	0.30	0.14
		100	−0.15	0.04	−0.02	0.19	−0.07	0.00
	H	25	−0.04	0.02	0.39	0.20	0.03	−0.08
		50	−0.10	0.02	0.24	0.21	0.03	0.02
		75	−0.11	0.01	0.60	0.31	0.28	−0.15
		100	−0.15	0.02	0.15	0.40	0.33	0.06
	R	25	−0.04	0.02	0.26	0.21	0.15	−0.02
		50	−0.12	0.01	0.25	0.28	0.20	0.02
		75	−0.10	0.04	0.52	0.30	0.18	−0.12
		100	−0.15	0.03	0.39	0.16	0.00	−0.13

SPAD—chlorophyll index; Fv/Fm—maximum quantum efficiency of PSII; A—photosynthetic rate; E—transpiration rate; gs—stomatal conductance; Ci—intercellular CO_2_ concentration; FS—flowering sunflower; RS—ripe sunflower; L + S—leaves and stems; H—heads; R—roots. The IRs showing inhibiting effects are marked with a gray background.

## Data Availability

The data that support the findings of this study are available from the corresponding author upon request.
